# Occupational, domestic and environmental mesothelioma risks in the British population: a case–control study

**DOI:** 10.1038/sj.bjc.6604879

**Published:** 2009-03-03

**Authors:** C Rake, C Gilham, J Hatch, A Darnton, J Hodgson, J Peto

**Affiliations:** 1 Institute of Cancer Research, Sutton, Surrey SM2 5NG, UK; 2 Epidemiology Unit, Health and Safety Executive, Bootle, Merseyside L20 3QZ, UK; 3 Department of Epidemiology and Population Health, London School of Hygiene and Tropical Medicine, London University, Keppel Street, London WC1E 7HT, UK

**Keywords:** mesothelioma, case–control, amosite

## Abstract

We obtained lifetime occupational and residential histories by telephone interview with 622 mesothelioma patients (512 men, 110 women) and 1420 population controls. Odds ratios (ORs) were converted to lifetime risk (LR) estimates for Britons born in the 1940s. Male ORs (95% confidence interval (CI)) relative to low-risk occupations for >10 years of exposure before the age of 30 years were 50.0 (25.8–96.8) for carpenters (LR 1 in 17), 17.1 (10.3–28.3) for plumbers, electricians and painters, 7.0 (3.2–15.2) for other construction workers, 15.3 (9.0–26.2) for other recognised high-risk occupations and 5.2 (3.1–8.5) in other industries where asbestos may be encountered. The LR was similar in apparently unexposed men and women (∼1 in 1000), and this was approximately doubled in exposed workers’ relatives (OR 2.0, 95% CI 1.3–3.2). No other environmental hazards were identified. In all, 14% of male and 62% of female cases were not attributable to occupational or domestic asbestos exposure. Approximately half of the male cases were construction workers, and only four had worked for more than 5 years in asbestos product manufacture.

The British mesothelioma death rate is now the highest in the world, with 1749 deaths in men (1 in 40 of all male cancer deaths below 80 years of age) and 288 in women in 2005. The projected lifetime risk (LR) of fatal mesothelioma in all British men born in the 1940s is 0.59% (Hodgson *et al*, 2005), or about 1 in 170 of all deaths. Substantial exposure to asbestos dust continued until about 1970 in parts of the asbestos industry, and until the early 1980s in the much larger workforce in construction and other occupations in which asbestos lagging was applied or asbestos insulation board (AIB) was sawn. The death rate is still increasing above 60 years of age, but the reduction in asbestos use since the mid-1970s has been followed 20 years later by a rapid fall in the number of mesothelioma deaths at 35–49 years of age in British men (289 in 1990–1994, 122 in 2000–2004), although less in women (56 in 1990–1994, 39 in 2000–2004).

Earlier case–control studies have used hospital-based controls or relied on next-of-kin interviews and most involved small numbers of cases (Muscat and Wynder, 1991; Howel *et al*, 1997; Teschke *et al*, 1997; Iwatsubo *et al*, 1998; Agudo *et al*, 2000; Rodelsperger *et al*, 2001). We carried out the first population-based study in Britain of the relationship between mesothelioma risk and lifetime occupational and residential history of asbestos exposure obtained at interview, and the largest worldwide. The main aim was to estimate risks and numbers of cases caused by specific occupational and environmental exposures. The results are particularly relevant to developing countries where uncontrolled asbestos exposure is still common.

## MATERIALS AND METHODS

Mesothelioma patients in England, Wales and Scotland born since 1925 were identified through chest physicians, surgeons and nurses, the National Cancer Research Network, and English and Scottish hospital records. A small proportion was identified through cancer registries and other sources. Population controls were selected at random from a 1 in 300 sample from Health Authority registers (now Primary Care Trusts), frequency matched by 5-year age group and sex. Owing to fears of legal liability under confidentiality and data protection law, no data were provided by any of the Health Boards in Scotland, 11 Health Authorities in England and 3 in Wales. Up to six randomly selected age-matched GP controls were approached for each interviewed mesothelioma case in these areas. The study was approved by the South Thames MREC.

Overall 39% of 1396 notified mesothelioma patients (423 too ill or dead, 87 no GP or consultant permission and 31 ineligible or not traced) and 18% of 2897 controls (169 too ill or dead, 169 no permission, 191 ineligible or untraced) were not invited for interview. The proportion of those invited who were interviewed, sometimes after several reminders, consisted of 73% (624 out of 857) of mesothelioma cases and 60% (1420 out of 2368) of controls. As expected, response rates in controls were higher in more affluent areas (69% in the top two quintiles of socioeconomic status and 46% in the lowest). There was no evidence that construction workers were under ascertained, with 8.3% of controls classified as working in skilled construction and building trades and 1.8% as carpenters, compared with 2001 census proportions of 6.5 and 1.7%, respectively. The study was initially restricted to cases born since 1940, later extended to 1925, to include those aged up to 80 years. This resulted in a higher proportion of younger cases than seen nationally (Table 1). Histological confirmation was obtained for 92% of interviewed mesothelioma cases.

Interviews were conducted between January 2001 and June 2006. Cases and controls were ineligible if they were physically or mentally unfit for interview, without access to a telephone or unable to speak English. All eligible patients were sent a pre-interview postal questionnaire requesting lifetime occupational and residential history. This formed the basis for the structured telephone interview, which also included questions on smoking history, DIY activities and other possible environmental exposures. Cases were asked about various asbestos exposures in each job, depending on the type of work. These included work with AIB, lagging, sprayed coatings, cement, insulation, heat-protection materials, gaskets, textiles and brake linings. For each job, the duration, description and occupational code were recorded, together with frequency of direct or bystander asbestos exposure.

### Statistical methods

Odds ratios (ORs) adjusted for 5-year period of birth and socioeconomic status (quintile of Townsend score from 2001 census data linked to postcode) were estimated by unconditional logistic regression analysis using STATA (StataCorp, 2007). Occupational histories were truncated in 1992. Occupations were assigned to risk categories primarily on the basis of proportional mortality ratios (PMRs) for mesothelioma (McElvenny *et al*, 2005). Non-construction high-risk occupations (most with PMR>200) included asbestos factory workers, laggers, shipbuilding and dockyard workers, naval personnel, and others working on board ships. Preliminary analysis showed that the risk was highest for carpenters and higher for plumbers, electricians and painters than for other construction workers. All other jobs were classified on the basis of Standard Occupational Classification (1990) code and main place of work as medium-risk industrial (primarily occupations with PMR>100, including mechanical and electrical process and assembly workers, welders, railway workers, surveyors, inspectors and industrial scientists), low-risk industrial (including motor mechanics, draughtsmen, warehousemen, drivers, plant and machine operators and armed forces) or non-industrial. Substantial asbestos exposure was defined as working with or disturbing AIB, sprayed coatings, lagging, other asbestos insulation or raw asbestos. Cases were thus classified into eight mutually exclusive exposure groups based on their highest exposure category irrespective of duration. These were ranked as (1) high-risk (including lagging and shipbuilding but excluding construction), (2) carpenter, (3) plumber, electrician, painter, (4) other construction, (5) medium-risk industrial, (6) substantial asbestos exposure in a low-risk occupation, (7) living with an occupationally exposed relative and (8) the remainder, comprising 52 cases (18 men, 34 women) and 439 controls (289 men, 150 women) with none of these exposures. Men who worked only in low-risk industrial or non-industrial jobs (groups 6, 7 and 8) constituted the reference group for the occupational analyses in Tables 3–5. Univariate analyses of domestic and neighbourhood exposures and work with other asbestos products (heat-protection materials, gaskets and brake linings) were restricted to cases who had only done low-risk industrial or non-industrial work not involving substantial exposure (groups 7 and 8). Possible non-occupational hazards included living with an asbestos worker or in a council property, prefab or high-rise block, living near a potentially hazardous site (asbestos factory or disposal site, power plant and shipyard) and DIY involving asbestos. A full report giving details of the occupational classifications and subgroup results will appear on the Health and Safety Executive's website (www.hse.gov.uk).

Lifetime risks in men were calculated from ORs as LR=OR × *A*÷*B*, where *A* is 0.59%, the predicted lifetime mesothelioma risk in British men born in the 1940s (Hodgson *et al*, 2005), and *B* is the weighted average OR in male controls. *B* equals 7.4 in Table 6, where the reference group is unexposed, and 5.0 in Tables 3–5, where the reference group includes men who reported domestic exposure or substantial occupational exposure in low-risk jobs.

## RESULTS

### Occupational exposure

**Figure 1:**
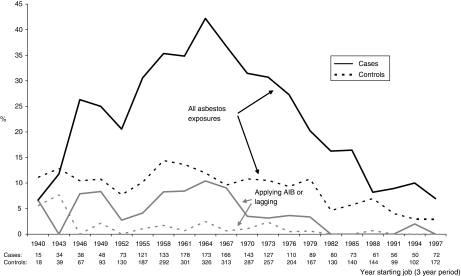
Proportion of all men reporting asbestos exposure by year starting new job.

Those interviewed (622 mesothelioma cases and 1420 controls) are shown by sex, period of birth and exposure category (ever/never worked in job groups 1–5) in Table 1. The occupational analyses in Tables 2, 3, 4 and 5 are restricted to men. Figure 1 shows the proportions of male cases and controls beginning a new job in each period since 1940 who reported asbestos exposure in that job. Jobs of more than 5 years duration are excluded to ensure that reported exposure is representative of the period when the job started. The frequency of exposure fell sharply during the 1970s, and the heaviest exposures (sawing AIB or applying lagging or sprayed coatings) had virtually ceased by 1982. The left-hand part of Table 2 shows ORs for male mesothelioma cases and controls who worked for at least 5 years in each main job group. Forty-six of the 102 non-construction high-risk mesothelioma cases had worked in docks, shipyards or on non-naval ships, and 26 in the Navy. The reference group in Table 2 comprises 25 cases and 278 controls with no high- or medium-risk or construction work and less than 5 years in low-risk industrial occupations. Carpenters suffered the highest risk (OR 36.0, 95% confidence interval (CI) 19.2–67.3), followed by all non-construction high-risk jobs (OR 16.8, 95% CI 9.6–29.3), plumbers, electricians and painters (OR 14.6, 95% CI 8.8–24.4) and other construction workers (OR 7.9, 95% CI 4.7–13.3). The overall OR for 5 or more years in any medium-risk industrial job was 5.2 (95% CI 3.3–8.2). The right-hand part of Table 2 shows the corresponding ORs for men who never worked in any higher risk category. The ORs for construction and medium-risk industrial workers remain high when those with higher risk occupations are excluded. In contrast, the OR for low-risk industrial occupations falls from 4.1 (95% CI 2.6–6.4) overall to 1.1 (95% CI 0.5–2.2) when those who had also worked in medium or higher risk jobs are excluded.

Table 3 shows ORs for men ever employed as carpenters (lower part) or in at least one high-risk, construction or medium-risk job (upper part) by age starting the first such job and duration of relevant employment. The OR in men first exposed before the age of 20 years is 13.4 (95% CI 9.2–19.6) for 20 or more years of any high-risk work, and 99.7 (95% CI 43.7–227.5) for 20 or more years as a carpenter. Analyses of duration before and after 30 years of age for all high-risk work (Table 4, upper part) and for carpentry (lower part) show a strong trend in OR with increasing duration only before 30 years of age. Odds ratios are consistently lower in each row in men who stopped exposure before 30 years of age, but there is no evidence of increasing risk with longer duration after 30 years, and the OR for men first employed in any high-risk job after 30 years was only 1.7 (95% CI 0.7–3.9).

In Table 5, men are assigned to the five occupational groups on the basis of the highest category in which they ever worked (see Materials and Methods). As in Tables 3 and 4, ORs are relative to men employed only in low-risk industrial or non-industrial jobs. The ORs and corresponding LRs for 10 or more years of relevant work before 30 years of age were highest for carpenters (OR 50.0, LR 5.9%), about one-third as high for electricians, plumbers and painters (OR 17.1, LR 2.0%) and for non-construction high-risk jobs (OR 15.3, LR 1.8%), and lower for other construction workers (OR 7.0, LR 0.8%) and for all medium-risk industrial jobs (OR 5.2, LR 0.6%). Odds ratios for all men in each category irrespective of duration are also shown, ranging from 23.3 for carpenters to 2.8 for medium-risk industrial workers.

The nature and frequency of reported substantial asbestos exposure were also predictive, with higher risks in each category in men reporting frequent direct exposure, but the OR was still significantly increased within each of the five exposure categories (Table 5) among men who did not report direct or indirect substantial exposure in any job before 30 years of age. The proportion of controls reporting direct exposure before 30 years of age was 67% (28 out of 42) among carpenters, 55% (63 out of 114) among plumbers, electricians and painters, 26% (37 out of 142) among other construction workers, 12% (32 out of 263) among medium-risk industrial workers and 3% (12 out of 413) among low-risk workers.

### Non-occupational exposure

The reference group in Tables 3, 4 and 5 included 33 men (7 cases, 26 controls) who reported substantial asbestos exposure in low-risk occupations. These were excluded in univariate analyses of other and non-occupational exposures, which were restricted to men (31 cases, 387 controls) and women (71 cases, 236 controls) who reported no high- or medium-risk work and no substantial exposure in any job. Other potential asbestos exposures at work were not associated with significantly increased risk, although few were reported. These included vehicle maintenance involving work with brakes or gaskets (OR 0.4, 95% CI 0.1–1.7), asbestos present at the workplace (OR 0.8, 95% CI 0.5–1.5), construction work at the workplace (OR 1.4, 95% CI 0.8–2.3), working in or near a shipyard or construction site (OR 1.3, 95% CI 0.3–6.1) and any other reported asbestos exposures at work (OR 1.6, 95% CI 0.7–3.6).

The only significant non-occupational association was living with a potentially exposed worker before 30 years of age (OR 2.0, 95% CI 1.3–3.2). The OR for living within a mile of a potential source before 30 years of age (asbestos factory or disposal site, shipyard or power plant) was 0.6 (95% CI 0.2–2.0). The OR for any type of DIY activity was 0.7 (0.4–1.2), and no subgroup of DIY activity by frequency or possible asbestos exposure suggested any excess. No type of housing was significantly associated with risk (prefab, council or former council, high rise or any asbestos in the building). The OR for current smokers compared with non-smokers was 1.5 (95% CI 0.8–2.7) in this unexposed subgroup, and 1.2 (95% CI 0.9–1.6) overall.

### Asbestos-related and background cases

All men and women are shown in the eight non-overlapping exposure categories (see Materials and Methods) in Table 6. Cases are classified by their highest exposure, so the groups are mutually exclusive. The reference group for this analysis comprises the 52 cases (18 men, 34 women) and 439 controls (289 men, 150 women) with none of these exposures. Five of the 110 female cases reported high-risk work (OR 4.8, 95% CI 1.3–17.7). A further 32 female cases had done medium-risk industrial work (OR 2.4, 95% CI 1.3–4.3), the majority (88%) as assemblers or routine process operatives. The risk was also increased in those who worked only in low-risk jobs but reported a substantial asbestos exposure (seven male and two female cases: combined OR 3.8, 95% CI 1.7–8.8). The ORs for domestic exposure before 30 years of age in Table 6 (2.1 for men, 1.9 for women; combined OR 2.0, 95% CI 1.3–3.2) are based on cases with no occupational exposure. Logistic regression analysis in all cases, adjusting for duration and main exposure group, gave an OR for domestic exposure before 30 years of age of 2.3 (95% CI 1.5–3.8) in women, significantly higher than the OR of 1.1 (95% CI 0.9–1.4) in men. Similar analyses based on the type of relative, irrespective of age, gave a combined estimate of 1.3 (95% CI 1.0–1.6) for living with a high-risk parent or sibling and an estimate of 2.1 (1.3–3.5) for living with a high-risk spouse. The latter was based largely on women, as only two male cases and one male control reported living with a high-risk spouse. Of controls, 20% reported living with a high-risk parent and 12% with a high-risk sibling, and 20% of female controls had lived with a high-risk spouse.

Table 6 shows the attributable fraction of cases in each category, ranging from 97% (86.4 out of 89) among male carpenters to 49% (24.3 out of 50) in men and women who lived with an exposed worker before 30 years of age. In all, 85% (435.6 out of 512) of male and 22% (24.5 out of 110) of female cases are attributed to occupational exposure, and a further 1.3% (6.8 out of 512) of male and 16% (17.5 out of 110) of female cases had no occupational exposure but were attributed to living with a high-risk worker. The total number of unattributed ‘background’ cases is 69.6 in men and 67.8 in women.

## DISCUSSION

Proportional mortality ratios for mesothelioma based on last full-time occupation as recorded on death certificates (McElvenny *et al*, 2005) provide the only estimates of occupational mesothelioma risks in Britain. The risk depends mainly on asbestos exposure below 30 years of age, however (Tables 3 and 4), and our study provide the first overview of the distribution of risk in the British population, the extraordinary risks suffered by men who did the most dangerous jobs when they were young and the contribution of environmental exposure, particularly to the families of exposed workers. Recall bias was minimised by structured questions about specific exposures, and our main analyses were based on job title, which should be less liable to differential response by cases than reported asbestos exposure. The value of lifetime job histories is illustrated by men who had worked for at least 5 years in low-risk industrial occupations; their ORs fell from 4.1 (95% CI 2.6–6.4) to only 1.1 (95% CI 0.5–2.2) when those who had also done more hazardous work were excluded (Table 2). Among motor mechanics, for example, only 2 of 18 cases compared with 23 of 54 controls had never worked in other more hazardous occupations.

Of male mesothelioma cases, 94% (481 out of 512) and 65% (725 out of 1112) of male controls had worked in a hazardous occupation (Table 6), implying an attributable fraction of 85%. The predicted lifetime mesothelioma risk for British men born in the 1940s who did more than 10 years of relevant work before 30 years of age is 5.9% for carpenters, 2.0% for plumbers, electricians and painters, and 0.8% for other construction workers (Table 5). The LR is 1.8% for all high-risk non-construction occupations, but this is a broad category that includes many subgroups with substantially higher risks. Table 2 shows their overall OR for 5 or more years’ work, which was 16.8 (95% CI 9.6–29.3), but the OR for metal plate workers (mainly shipbuilders) was 43.3 (95% CI 13.5–138.6). Although this is the largest published study of mesothelioma risk in relation to lifetime occupational history, much larger numbers would be needed to provide reliable estimates for individual occupations.

The proportion of men who had ever worked in carpentry was 4% of controls and 21% of mesothelioma cases (Table 4: 49 out of 1112 controls, 105 out of 512 cases), and 33% (10 out of 30) of mesothelioma cases in men born since 1950 who started work in 1970 or later, so the predicted eventual total of 90 000 mesothelioma deaths in both sexes in Britain by 2050 (Hodgson *et al*, 2005) will include about 15 000 former carpenters. The excess lung cancer risk in heavily exposed workers is likely to be of the same order as the mesothelioma risk (Darnton *et al*, 2006), so more than 1 in 10 of all British carpenters born in the 1940s may die of a cancer caused by asbestos. A substantial proportion of these deaths will be among those who installed AIB as fireproofing required under building regulations (HMSO, 1965) and these will far exceed any possible benefit of such work to the public. The large amount of asbestos that remains in many older buildings is still a potential hazard to construction workers involved in renovation, maintenance or asbestos removal.

The risk to carpenters is particularly high in Britain, and also in Australia (Yeung *et al*, 1999). Most studies in other countries reported higher risks in plumbers, electricians and other construction workers than in carpenters (Muscat and Wynder, 1991; Teschke *et al*, 1997; Rodelsperger *et al*, 2001). Among construction workers, preliminary results of a French population-based case–control study show the highest risks in plumbers, pipe fitters, structural steel workers and construction labourers (Goldberg *et al*, 2006). In a large non-interview study in California based on main occupation of over 2000 mesothelioma cases and pancreatic cancer controls, increased ORs were seen for plumbers (OR=4.9, 95% CI 2.8–8.3), electricians (OR=3.8, 95% CI 2.0, 7.1) and painters (OR=2.6, 95% CI 1.3–5.3) but not for carpenters (OR=1.2, 95% CI 0.8–2.0) (Pan *et al*, 2005).

The ORs within each occupational exposure category were lower but still substantially increased even in men who recalled no substantial asbestos exposure, suggesting that many were exposed indirectly or could not identify the asbestos materials they handled. Most people report their own and their parents’ occupations correctly many years later (Berney and Blane, 1997), but recall of past asbestos exposure shows poor reproducibility at re-interview (Holmes and Garshick, 1991). A recent study of plumbers showed that many do not recognise the friable asbestos materials that they still sometimes encounter (Bard and Burdett, 2007). The increased risk for medium-risk industrial work reflects widespread and often unrecognised contact with asbestos in metal working, electrical trades and assembly line work. A large proportion of the British population worked in these sectors (24% of male and 20% of female controls). There were too few occupationally exposed female mesothelioma cases to estimate risks reliably, but Table 6 suggests that workplace exposure caused about 22% (24.5 out of 110) of all female cases. The occupational hazard in women was concentrated in medium-risk industrial settings, particularly assembly line work. The only evidence of an occupational risk outside high- or medium-risk and construction occupations was the seven men and two women who reported substantial personal or bystander exposure in other occupations (Table 6).

### Non-occupational exposure

The only substantial risk factor in those with no direct occupational exposure was living with a high-risk worker, a hazard that has been recognised for many years (Newhouse and Thompson, 1965; Vianna and Polan, 1978; Joubert *et al*, 1991; Bourdes *et al*, 2000; Magnani *et al*, 2001). The excess risk, which was confined to those who lived with an exposed worker before 30 years of age and was similar in men and women (combined OR 2.0, 95% CI 1.3–3.2), corresponds to an increase in LR of about 1 per 1000. There was no overall risk in men and women who reported living within a mile of a potential environmental hazard (asbestos factory or disposal site, shipyard or power plant) before 30 years of age, although the risk was non-significantly increased in those who lived near one of these potential sources for 20 or more years (OR 3.3, 95% CI 0.7–14.8, based on three cases and six controls). We had no means of identifying sites that produced substantial local exposure. There was certainly some hazard to residents around a few factories in the past (Newhouse and Thompson, 1965; Magnani *et al*, 2001), but no risk was seen around others (Hammond *et al*, 1979), and many apparently environmental cases are related to occupational exposures (Arblaster *et al*, 1995). There was no significant difference between current smokers and lifelong non-smokers either in this apparently unexposed subgroup (OR 1.5, 95% CI 0.8–2.7) or overall (OR 1.2, 95% CI 0.9, 1.6).

The unattributed cases in each row of Table 6 are presumably due to ambient or unreported asbestos exposure or to other or natural causes. This unexplained risk is similar for non-industrial (including retail, office, educational, health-care and agricultural) and low-risk industrial work, and corresponds to a predicted LR of 0.08% in men. The analysis of attributable risk in Table 6 indicates that these background cases accounted for 69.6 (14%) of the 512 male and 67.8 (62%) of the 110 female mesothelioma cases. The same analysis restricted to cases born during 1940–1949 also gave similar male and female estimates for the number of unattributed cases (31.9 out of 274 in men and 36.7 out of 51 in women). Table 1 shows that the male/female ratio of interviewed cases born during 1940–1949 (5.4 : 1) was close to that of all British mesothelioma deaths during 2000–2004 among those born during 1940–1949 (5.7 : 1). The annual number of unexplained mesothelioma cases is thus similar in men and women, and corresponds to a LR of the order of 1 per 1000 among Britons of both sexes born in the 1940s.

The cumulative female mesothelioma death-rate by age 70 is now more than three times higher in the UK (0.037%) than in the US (0.012%). If this is due to differences in asbestos exposure, more than two thirds of mesotheliomas in British women born since the 1930s are caused by asbestos, far more than the 38% (Table 6: 42.0/110) that were attributed to identified exposures in our study. A similar conclusion is suggested by the three-fold increase in the death-rate in British women between 1975–1979 and 2000–2004. This would imply that at least 30% of female cases (of the order of 100 per year) are caused either by environmental asbestos exposure or by occasional or ambient exposure in occupational settings that we have classified as low risk. If so, there is presumably a similar number in men. Many apparently spontaneous mesotheliomas are therefore likely to be due to an increase in ambient asbestos exposure that coincided with the widespread occupational exposures of the 1960s and 1970s.

### Future trends

The upper part of Table 4 shows that high- or medium-risk exposure beginning after 30 years of age did not cause a statistically detectable increase in risk. Among men already exposed for 10 or more years before 30 years of age, however, a further 10 or more years of exposure after 30 years of age increased the OR from 6.8 to 13.1 for all high-risk work. The estimated factor is 2.1 (95% CI 1.1–4.0) in an analysis restricted to these two subgroups, suggesting that at 30 years of age, the mesothelioma risk is still increased substantially by continuing asbestos exposure. The future burden of mesothelioma is thus still uncertain. The death rate in men born around 1945 is higher up to 55 years of age than in any earlier or later birth cohort, but few had substantial asbestos exposure after 30 years of age, and we still do not know how rapidly their mortality will increase at older ages.

### Contribution of amosite

The mesothelioma risk caused by amosite (brown asbestos) is two orders of magnitude greater than that by chrysotile (white asbestos) (Hodgson and Darnton, 2000), and it seems likely that a major cause of the extraordinary risk to British carpenters was cutting amosite board with power tools, which was widespread in the UK construction industry through the 1970s and continued into the 1980s. A comparison of current mesothelioma death rates and imports to the US (Virta, 2006) and UK (MRC Institute for Environment and Health, 1997) of white, brown and blue (crocidolite) asbestos also suggests that the much higher mesothelioma death-rate in the UK was caused by its much greater use of amosite. The mesothelioma death-rate in men aged 45–49 is now more than three times higher in the UK than in the US (US: 0.26 per 100 000 in 2000–2004, based on 139 deaths; UK: 0.87 per 100 000 in 2001–2004, based on 66 deaths). These men were born between 1950 and 1959, and few would have had much asbestos exposure before 1970. The UK used slightly less chrysotile during the 1970s (2.4 kg per head in 1970, 1.7 in 1980) than the US (3.1 kg per head in 1970, 1.5 in 1980), and the UK had virtually ceased using crocidolite by 1970, while US crocidolite imports increased from 8 900 tonnes in 1970 to a peak of 16 900 tonnes (0.08 kg per head) in 1978 and remained above 5 000 tonnes per year until 1984. However, the UK imported far more amosite per head than the US in the 1970s. UK amosite imports were 21 600 tonnes (0.4 kg per head) in 1970 and did not decline until after 1976, while US amosite imports fell from 12 900 tonnes (0.07 kg per head) in 1970 to 1 400 tonnes in 1976 (MRC Institute for Environment and Health, 1997; Virta, 2006). Australia's mesothelioma death-rates are similar to Britain's, and so were its patterns of amosite and crocidolite use (Leigh and Driscoll, 2003).

The UK control limit remained the same for amosite as for chrysotile (BOHS, 1973) until 1983, when it was reduced from 2 to 1 fml^−1^ for chrysotile and from 2 to 0.5 fml^−1^ for amosite. The 2 fml^−1^ control limit was probably observed in most asbestos factories during the 1970s, and only four of the 512 male mesothelioma cases in our study had worked for more than 5 years in asbestos product manufacturing; but substantial asbestos exposure was widespread in the much larger workforce in construction.

## Figures and Tables

**Table 1:** Interviewed mesothelioma cases and controls by job category and all British mesothelioma deaths, by year of birth and sex

		**Mesothelioma cases**				**Population controls**				**National mesothelioma register 2000–2004**	
**Year of birth**	**Central age^a^**	**Ever worked in high-risk jobs^b^**	**Never worked in high-risk jobs^c^**	**Total**	**%**	**Ever worked in high-risk jobs^b^**	**Never worked in high-risk jobs^c^**	**Total**	**%**	***n***	**%**
*Male cases*											
⩾1965	35	1	1	2	0.4	1	3	3	0.3	3	0.1
1960–1964	40	4	0	4	0.8	5	7	13	1.2	14	0.2
1955–1959	45	11	2	13	2.5	20	16	36	3.2	48	0.8
1950–1954	50	42	2	44	8.6	66	45	111	10.0	166	2.9
1945–1949	55	105	14	119	23.2	162	123	285	25.6	524	9.0
1940–1944	60	145	10	155	30.3	252	128	380	34.2	893	15.3
1935–1939	65	82	7	89	17.4	97	46	143	12.9	1223	21.0
1930–1934	70	48	1	49	9.6	52	27	79	7.1	1420	24.4
1925–1930	75	36	1	37	7.2	44	18	62	5.6	1529	26.3
Total		474	38	512	100.0	699	413	1112	100.0	5820	100.0
											
*Female cases*											
⩾1965	35	0	2	2	1.8	0	0	0		5	0.6
1960–1964	40	0	1	1	0.9	2	4	6	1.9	5	0.6
1955–1959	45	1	7	8	7.3	1	13	14	4.5	23	2.6
1950–1954	50	2	5	7	6.4	7	13	20	6.5	31	3.4
1945–1949	55	6	11	17	15.5	9	45	54	17.5	86	9.5
1940–1944	60	13	21	34	30.9	17	75	92	29.9	162	18.0
1935–1939	65	8	17	25	22.7	22	55	77	25.0	181	20.1
1930–1934	70	5	7	12	10.9	9	21	30	9.7	178	19.8
1925–1930	75	2	2	4	3.6	4	11	15	4.9	230	25.5
Total		37	73	110	100.0	71	237	308	100.0	901	100.0

a In all, 97% (601 out of 622) of cases were diagnosed between 2000 and 2004.

b Ever worked in job categories 1–5: non-construction high-risk, carpenter, plumber/electrician/painter, other construction and medium-risk industrial jobs.

c Worked only in low-risk industrial or non-industrial jobs.

**Table 2:** Numbers of male mesothelioma cases and controls who worked for at least 5 years before 1992 in each occupational category

**Occupational category**	**Cases**	**Controls**	**OR (95% CIs)**	**Cases**	**Controls**	**OR (95% CIs)**
*Non-construction high risk*						
Any non-construction high-risk job	102	78	16.8 (9.6, 29.3)			
						
*Construction*				*No other high-risk work*		
Carpenter	93	36	36.0 (19.2, 67.3)	81	30	39.3 (20.2, 76.5)
Plumber, electrician, painter or decorator	115	96	14.6 (8.8, 24.4)	99	82	14.8 (8.7, 25.1)
Other construction	81	120	7.9 (4.7, 13.3)	59	101	6.8 (4.0, 11.7)
						
*Medium-risk industrial*				*No high-risk or construction work*		
Any medium-risk industrial job	157	331	5.2 (3.3, 8.2)	57	201	3.2 (1.9, 5.3)
						
*Low-risk industrial*				*No high-risk, construction or medium-risk work*		
Any low-risk industrial job	153	406	4.1 (2.6, 6.4)	13	135	1.1 (0.5, 2.2)
Reference group^a^	25	278	1.0 (ref)	25	278	1.0

Abbreviations: CI=confidence interval; OR=odds ratio.

Men with any exposure in preceding occupational categories are excluded in the right-hand part of the table.

a The reference group worked only in non-industrial jobs or in low-risk industrial jobs for less than 5 years.

**Table 3:** Odds ratios for male cases by age started and total duration in high-risk^a^ occupations and in carpentry

	**Duration of employment**									
	**<5 years**		**5–9 years**		**10–19 years**		**⩾20 years**		**Total**	
**Age at first job of this type**	**Cases/ controls**	**OR (95% CI)**	**Cases/ controls**	**OR (95% CI)**	**Cases/ controls**	**OR (95% CI)**	**Cases/ controls**	**OR (95% CI)**	**Cases/ controls**	**OR (95% CI)**
*All high-risk jobs* a										
<20	27/75	4.0 (2.3, 6.9)	36/77	5.2 (3.1, 8.7)	51/93	5.9 (3.6, 9.6)	311/265	13.4 (9.2, 19.6)	425/510	9.2 (6.4, 13.1)
20–29	7/41	1.8 (0.8, 4.4)	3/16	2.0 (0.6, 7.3)	7/37	2.1 (0.9, 5.0)	24/46	5.5 (3.0, 10.1)	41/140	3.1 (1.9, 5.0)
⩾30	5/15	3.6 (1.2, 10.4)	0/6	0.0 (0.0, 7.1)^b^	2/23	1.0 (0.2, 4.3)	1/5	1.8 (0.2, 16.3)	8/49	1.7 (0.7, 3.9)
Total	39/131	3.2 (2.0, 5.3)	39/99	4.3 (2.6, 7.2)	60/153	4.2 (2.7, 6.6)	336/316	12.1 (8.3, 17.6)	474/699	7.3 (5.1, 10.4)
										
*Carpentry*										
<20	7/9	9.0 (3.0, 26.4)	6/7	10.0 (3.0, 33.1)	14/5	38.9 (12.4,122.1)	63/10	99.7 (43.7,227.5)	90/31	38.0 (21.3, 67.5)
⩾20	5/4	21.3 (4.8, 93.4)	2/3	9.3 (1.4, 61.9)	3/6	5.5 (1.3, 24.5)	5/5	11.0 (2.9, 41.1)	15/18	10.0 (4.6, 21.9)
Total	12/13	11.0 (4.5, 27.0)	8/10	9.2 (3.3, 25.9)	17/11	18.5 (7.8, 44.1)	68/15	62.5 (30.9,126.5)	105/49	26.5 (15.9, 44.2)

Abbreviations: CI=confidence interval; OR=odds ratio.

Odds ratios are relative to the 38 cases and 413 controls with no high-risk, construction or medium-risk industrial jobs.

a High risk includes job categories 1–5: non-construction high-risk, carpenter, plumber/electrician/painter, other construction and medium-risk industrial jobs.

b Unadjusted Cornfield confidence interval.

**Table 4:** Odds ratios for male cases by duration of employment in high-risk^a^ occupations and in carpentry before and after 30 years of age

	**Duration after 30 years of age**							
	**None**		**<10 years**		**⩾10 years**		**Total**	
**Duration before 30 years of age**	**Cases/ controls**	**OR (95% CI)**	**Cases/ controls**	**OR (95% CI)**	**Cases/ controls**	**OR (95% CI)**	**Cases/ controls**	**OR (95% CI)**
*All high-risk jobs* a								
None			5/21	2.5 (0.9, 6.8)	3/28	1.1 (0.3, 3.8)	8/49	1.7 (0.7, 3.9)
<10 years	59/163	3.9 (2.5, 6.1)	27/76	3.9 (2.2, 6.8)	47/104	4.7 (2.9, 7.7)	133/343	4.1 (2.8, 6.1)
⩾10 years	19/31	6.8 (3.5, 13.3)	54/53	11.5 (6.9, 19.1)	260/223	13.1 (8.9, 19.3)	333/307	12.1 (8.4, 17.6)
Total	78/194	4.4 (2.8, 6.7)	86/150	6.2 (4.1, 9.6)	310/355	9.6 (6.6, 14.0)	474/699	7.3 (5.1, 10.4)
								
*Carpentry*								
None			3/7	6.5 (1.5, 28.0)	2/4	7.0 (1.2, 42.2)	5/11	6.6 (2.1, 21.0)
<10 years	16/16	11.4 (5.1, 25.6)	6/1	58.3 (6.4, 532.1)	8/6	15.1 (4.8, 47.2)	30/23	14.6 (7.6, 28.3)
⩾10 years	7/3	26.3 (6.3,109.7)	12/2	93.1 (18.9, 458.3)	51/10	73.8 (32.3, 168.9)	70/15	66.3 (32.5, 135.5)
Total	23/19	13.4 (6.5, 27.6)	21/10	28.1 (11.6, 67.8)	61/20	40.9 (21.3, 78.8)	105/49	26.5 (15.9, 44.2)

Abbreviations: CI=confidence interval; OR=odds ratio.

Odds ratios are relative to the 38 cases and 413 controls with no high-risk, construction or medium-risk industrial jobs.

a High risk includes job categories 1–5: non-construction high-risk, carpenter, plumber/electrician/painter, other construction and medium-risk industrial jobs.

**Table 5:** Odds ratios for male cases by duration of employment before 30 years of age for job category and reported asbestos exposure

		**Construction**			**All other jobs**	
**Duration in job category before 30 years of age**	**Non-construction high risk**	**Carpenters**	**Plumbers, electricians, painters**	**Other construction**	**Medium-risk industrial**	**Low risk (ref)**
*None*						
Cases/controls	4/15	3/10	6/12	7/24	4/26	
OR (95% CI)	2.8 (0.9, 8.9)	3.1 (0.8, 11.8)	5.2 (1.8, 14.9)	3.2 (1.3, 8.1)	1.5 (0.5, 4.6)	
						
*<5 years*						
Cases/controls	52/59	9/12	13/34	20/66	15/79	
OR (95% CI)	9.8 (5.9, 16.2)	8.2 (3.2, 20.9)	4.0 (1.9, 8.3)	3.3 (1.8, 6.0)	2.1 (1.1, 4.0)	
						
*5–9 years*						
Cases/controls	42/25	11/5	24/22	12/30	8/68	
OR (95% CI)	18.3 (10.0, 33.7)	24.4 (8.0, 74.9)	12.3 (6.3, 24.2)	4.3 (2.0, 9.0)	1.2 (0.6, 2.8)	
						
*⩾10 years*						
Cases/controls	55/39	66/15	69/46	13/22	41/90	
OR (95% CI)	15.3 (9.0, 26.2)	50.0 (25.8, 96.8)	17.1 (10.3, 28.3)	7.0 (3.2, 15.2)	5.2 (3.1, 8.5)	
Lifetime risk for ⩾10 years duration^a^	1.8%	5.9%	2.0%	0.8%	0.6%	0.12%
						
*Reported asbestos exposure before 30* years of age^b^						
*None*						
Cases/controls	29/56	12/12	16/38	23/91	35/202	33/393
OR (95% CI)	5.5 (3.1, 9.7)	9.8 (4.1, 23.5)	4.4 (2.2, 8.7)	2.7 (1.5, 4.8)	1.9 (1.1, 3.1)	
*Indirect*^c^						
<5 years						
Cases/controls	5/14	2/1	2/4	4/6	4/16	1/1
OR (95% CI)	4.0 (1.3, 11.7)	16.8 (1.4, 196.0)	5.0 (0.9, 28.9)	7.6 (2.0, 28.5)	2.5 (0.8, 7.9)	
⩾5 years						
Cases/controls	23/11	1/1	15/9	4/8	6/13	1/7
OR (95% CI)	21.7 (9.7, 48.5)	10.2 (0.6, 171.2)	18.3 (7.4, 44.9)	6.1 (1.7, 21.4)	4.7 (1.7, 13.2)	
*Direct infreq*^d^						
<5 years						
Cases/controls	10/6	11/11	5/19	2/7	1/8	0/4
OR (95% CI)	18.0 (6.1, 53.2)	12.1 (4.9, 30.0)	2.9 (1.0, 8.2)	3.0 (0.6, 15.1)	1.2 (0.1, 9.8)	
⩾5 years						
Cases/controls	20/16	32/8	39/25	8/17	11/18	1/7
OR (95% CI)	13.5 (6.4, 28.5)	49.6 (21.1, 116.4)	17.4 (9.5, 32.0)	5.5 (2.2, 13.6)	6.9 (3.0, 15.8)	
*Direct freq*^e^						
<5 years						
Cases/controls	26/13	12/4	6/7	4/7	2/1	1/1
OR (95% CI)	22.3 (10.5, 47.4)	32.0 (9.7, 105.5)	9.5 (3.0, 29.9)	6.4 (1.8, 23.0)	22.4 (1.9, 261.3)	
⩾5 years						
Cases/controls	40/22	19/5	29/12	7/6	9/5	1/0
OR (95% CI)	19.2 (10.2, 36.1)	38.4 (13.5, 109.6)	27.5 (12.9, 58.6)	11.0 (3.4, 34.9)	20.7 (6.5, 65.5)	
						
*Ever worked in job category*						
Cases/controls	153/138	89/42	112/114	52/142	68/263	38/413
OR (95% CI)	11.9 (7.9, 18.0)	23.3 (14.1, 38.5)	10.8 (7.1, 16.5)	4.0 (2.5, 6.4)	2.8 (1.8, 4.3)	1.0 (ref)

Abbreviations: CI=confidence interval; OR=odds ratio.

Cases who ever worked in each job category are excluded in subsequent columns.

a Projected lifetime risk to age 90=OR × 0.59%÷5.0. See statistical methods.

b Type and duration of asbestos exposure in any job category. Groups are mutually exclusive that is if several exposures occur, these are coded hierarchically.

c Work done by someone in the same area.

d Work done themselves less than once per week.

e Work done themselves at least weekly.

**Table 6:** Mesothelioma cases in men and women attributed to asbestos exposure in each exposure category

	**Male cases**					**Female cases**				
	**Cases**					**Cases**				
	**Attributed to this exposure**					**Attributed to this exposure**				
**Highest exposure category**	**Yes**	**No^a^**	**Total**	**Controls**	**OR (95% CI)**	**Yes**	**No^a^**	**Total**	**Controls**	**OR (95% CI)**
*Occupational exposure*										
Non-construction high risk	144.3	8.7	153	138	17.5 (10.3, 29.8)	4.0	1.0	5	5	4.8 (1.3, 17.7)
Carpenters	86.4	2.6	89	42	34.2 (18.7, 62.6)	—	—	0	0	—
Plumbers, electricians and painters	105.0	7.0	112	114	15.9 (9.2, 27.3)	—	—	0	2	—
Other construction	43.2	8.8	52	142	5.9 (3.3, 10.5)	—	—	0	1	—
Medium-risk industrial	51.4	16.6	68	263	4.1 (2.4, 7.2)	18.7	13.3	32	63	2.4 (1.3, 4.3)
Other substantial exposure	5.3	1.7	7	26	4.2 (1.6, 10.9)	1.8	0.2	2	1	9.6 (0.8, 112.3)
										
*Non-occupational exposure*										
Domestic exposure before 30 years of age	6.8	6.2	13	98	2.1 (1.0, 4.5)	17.5	19.5	37	86	1.9 (1.1, 3.2)
None of the above (reference)	—	18.0	18	289	1.0	—	34.0	34	150	1.0
Total	442.4	69.6	512	1112	7.4^b^	42.0	67.8	110	308	1.6^b^

Abbreviations: CI=confidence interval; OR=odds ratio.

Cases in each row are excluded in subsequent rows.

a Number not attributed to exposure=total cases/OR.

b Average of ORs weighted by number of controls in each row.
